# Safety and effectiveness of 4-week therapy with aceclofenac controlled release once a day

**DOI:** 10.1038/s41598-022-20633-6

**Published:** 2022-10-03

**Authors:** Ju-cheol Jeong, Yoon Hee Chung, Taejun Park, Seung Yeon Park, Tae Woo Jung, A. M. Abd El-Aty, Joon Seok Bang, Ji Hoon Jeong

**Affiliations:** 1 Department of Pharmacology, College of Medicine, Chung-Ang University, Seoul, 06974 Republic of Korea; 2 Clinical Research Team, Korea United Pharm Inc., Seoul, Republic of Korea; 3 Department of Anatomy, College of Medicine, Chung-Ang University, Seoul, Republic of Korea; 4 Department of Global Innovative Drug, The Graduate School of Chung-Ang University, Seoul, Republic of Korea; 5 Department of Pharmacology, Faculty of Veterinary Medicine, Cairo University, Giza 12211, Egypt; 6 Department of Medical Pharmacology, Medical Faculty, Ataturk University, Erzurum 25240, Turkey; 7 College of Pharmacy, Sookmyung Women’s University, Seoul, Republic of Korea

**Keywords:** Medical research, Rheumatology

## Abstract

Aceclofenac controlled-release (CR) is a once-a-day tablet with 200 mg of aceclofenac, and is bioequivalent to conventional aceclofenac. However, its safety in humans has not been well studied in Korea. Therefore, we aimed to evaluate the overall incidence and patterns of adverse events (AEs), the effectiveness of aceclofenac CR, and the differences in incidence rates of the AEs based on each patient’s baseline charateristics. This study was conducted on patients receiving aceclofenac CR in clinical practice at each investigational institution to treat musculoskeletal pain and inflammation. The subjects were administered one tablet of aceclofenac CR (200 mg once-a-day) and were observed for 4 weeks post-administration. Factors affecting the occurrence of AEs were evaluated, and the Visual Analogue Scale (VAS) was used to measure the pain intensity. Among 14,543 subjects, the incidence rate of AEs was 0.86%, and that of adverse drug reactions was 0.74%. No serious AEs and unexpected adverse drug reactions were monitored. The incidence rates of AEs were significantly higher in females, inpatient treatment, individuals with concurrent disorders, and those receiving concomitant medications, respectively (all *P* < 0.05). Four weeks post-using aceclofenac CR, the mean changes in VAS was significantly decreased compared to prior administration. The overall clinical efficacy rate was 91.63%. This study confirmed that no severe adverse reactions were observed for aceclofenac CR exceeding those previously reported for safety results of conventional formulation of this drug in routine clinical practice settings. The use of aceclofenac CR might not violate the previously reported information on the safety and effectiveness of aceclofenac.

## Introduction

Nonsteroidal anti-inflammatory drugs (NSAIDs) are usually recommended as a first-line treatment for mild to moderate pain and inflammation^1,2^. NSAIDs block cyclooxygenase (COX) enzymatic activity, which is involved prostaglandins (PGs) synthesis, and exhibit anti-inflammatory effects^2–4^. COX-1 inhibition leads to gastrointestinal toxicity, such as gastric erosions, ulcers, and mucosal bleeding. These undesirable effects limit the use of NSAIDs, with a substantial proportion of patients needing outpatient or inpatient palliative care possibly ending with death^5–10^.

Aceclofenac has a relatively high selectivity for COX-2 and site-specific inflammation^11–13^. Postmarketing surveillance of the UK drug monitoring system reported adverse reactions for aceclofenac during the first year after marketing. The incidence of gastrointestinal bleeding, abdominal pain, and hypertension was low. The overall incidence of adverse reactions was also significantly lower than that of meloxicam and rofecoxib^14^.

Aceclofenac controlled-release (CR) is a once-a-day tablet with 200 mg of aceclofenac as its active ingredient. A sustained-release formulation can improve adherence and clinical outcomes while benefiting from its fast-acting properties^15^. Aceclofenac CR is bioequivalent to conventional aceclofenac (100 mg twice daily)^15–17^. However, its safety in humans has not been well studided. Therefore, we aimed to verify the incidence of adverse effects of aceclofenac CR in Korean patients through collecting already known adverse effects (from the existing aceclofenac formulation) and all harmful cases not reported in prior studies concerning the use of aceclofenac CR tablet, and to assure the effectiveness of this formulation in a suitable medical environment.

## Methods

This study was conducted at multiple sites, including university hospitals, general hospitals, public health centers, and private local clinics in the Republic of Korea. From 2010 to 2013, the medical records of those outpatients receiving aceclofenac CR at each hospital or clinic were collected and continuously investigated without omission. This study was conducted in accordance with the Declaration of Helsinki and International Conference on Harmonisation Guideline for Good Clinical Practice.

### Study subjects

This study included outpatients expected to take aceclofenac CR for more than 4 weeks in routine clinical practice settings. All patients who were initially administered aceclofenac CR tablet for rheumatoid arthritis, ankylosing spondylitis, osteoarthritis, scapulohumeral periarthritis, lumbago, ischiadynia, and pain caused by nonarticular rheumatism were included^18–22^. Patients with active peptic ulcer or bleeding, or a history of that disease, those who have hypersensitivity to some drugs with the same ingredients or in the same class (such as diclofenac), and those who have a history of asthma, urticaria or allergic reaction to aspirin or other NSAIDs were excluded. Further, patients with severe heart failure, severe renal or hepatic impairments, inflammatory bowel disease, bleeding or coagulation disorders, those with a previous history of gastrointestinal bleeding or perforation (due to NSAIDs), and pregnant and lactating women were excluded from this study.

Although this observational study collected only information on the medical behaviour and treatment results, we obtained an approval of institutional review board (IRB) to conduct ethically to protect human rights with informed consent. The overall clinical protocol with consent forms were approved by IRB of Seoul National University Hospital (No. H-1207-146-420). In case of exemption from obtaining the consent forms, the exemption protocol was approved by IRB of Chung-Ang University Hispital (No. C2012183(878)).

### Safety and effectiveness assessments

In practice, one tablet of aceclofenac CR 200 mg (Clanza^®^ CR, Korea United Pharmaceutical Co., Seoul, Republic of Korea) was administered to all subjects participating in this study once a day, without chewing or crushing^15,18^. All adverse events (AEs) during 4 weeks of taking aceclofenac CR were recorded regardless of the causal relationship through a medical exam at Week 4. All undesirable changes noticed by the clinician and all AEs caused by this drug were included. The occurrence of harmful cases was classified according to the System Organ Class (SOC) and Preferred Term (PT) of the World Health Organization-Adverse Reactions Terminology (WHO-ART)^23,24^. The detected AEs were categorized based on the degree and seriousness of the AE, and the number of occurrences and percentages of each were calculated, respectively. To identify factors that might affect safety, the incidence rate for each factor among the subjects was evaluated. In addition, the symptoms, signs, and difference in pain intensity measured by 10 cm-VAS (Visual Analogue Scale) prior to and after administration were compared and evaluated^25–27^. The practical clinical rate of the patients was obtained by taking into careful consideration the patient’s subjective and objective symptoms, signs and 10 cm-VAS measurement results.

### Statistical analysis

This study presents the mean ± standard deviation, median, minimum, and maximum values for continuous data variables, while the frequency and ratios are presented for categorical data variables. A 95% confidence interval for the number and rate of incidence of the AEs and adverse drug reactions were summarized. The number and rate of incidence of AEs according to the characteristics of individual factors were presented and analyzed using Pearson's chi-square test or Fisher's exact test to verify the difference according to individual factors. Multivariable logistic regression analysis was used to estimate the factors, including gender, medical treatment, gastrointestinal disturbance, concomitant diseases, and concomitant medications, which could affect the occurrence of AEs. SAS ver. 9.3 (SAS Institute, Cary, NC, USA) was used for statistical analysis. For all *P*-values, < 0.05 was regarded as statistically significant.

### Ethics approval and consent to participate

This study was conducted ethically to protect human rights. It is an observational investigation that collects only information on the medical behaviour and treatment results. Therefore, the exemption from obtaining the consent forms for subjects information was approved by the institutional review board (IRB). However, if the IRB did not approve the exemption, the consent forms were obtained from the subjects prior to conducting this study.

## Results

**Figure 1:**
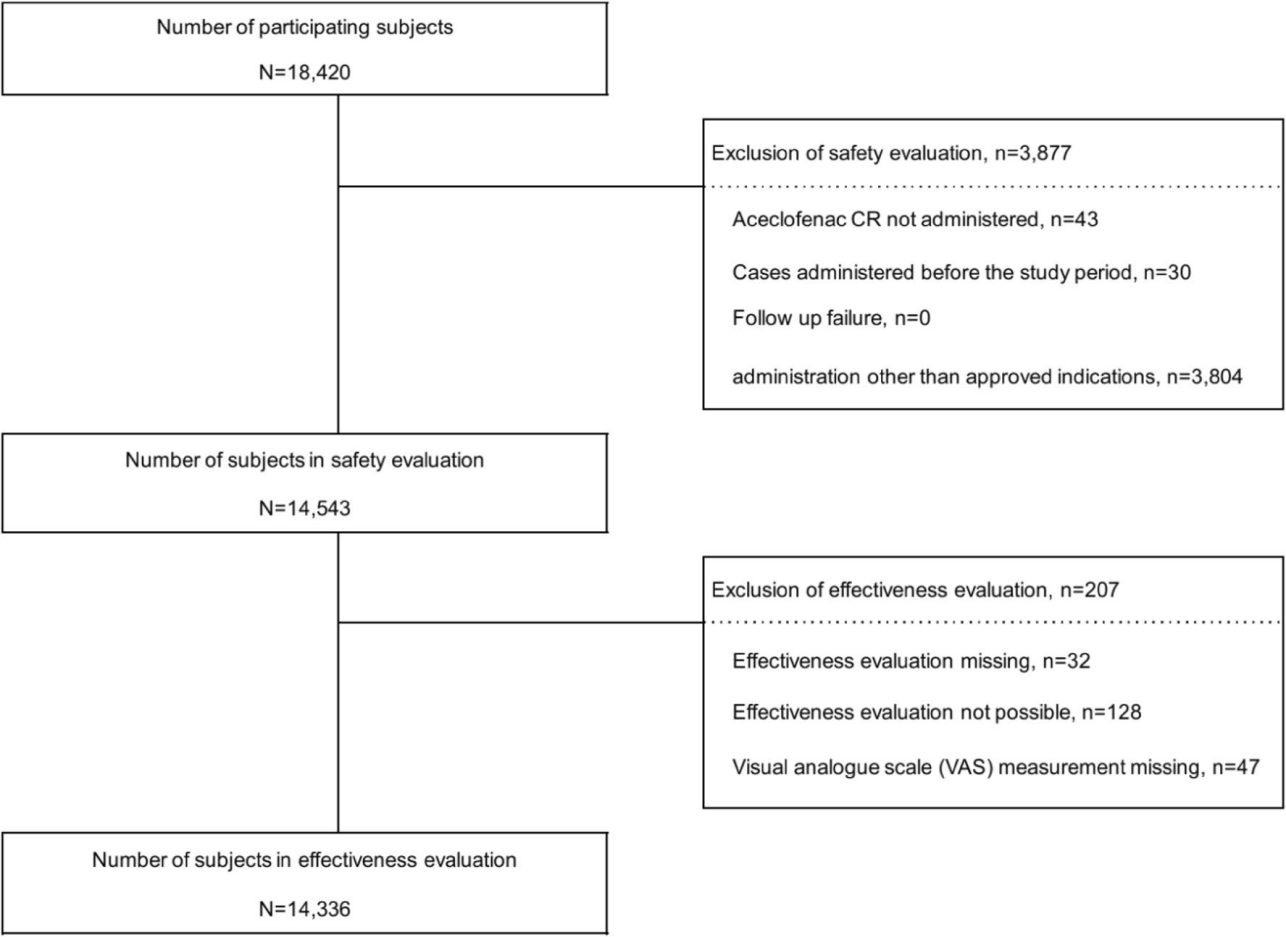
Participation status of the study subjects.

A total of 18,420 subjects from 487 institutions have participated in this study. Among them, 14,543 subjects were included in the safety evaluation analysis. Other, 3877 participants who were not taken aceclofenac CR, given aceclofenac CR before the conclusion of study contracts, or taken aceclofenac CR for a use other than the approved indications were excluded. Among the participants for safety evaluation, a total of 14,336 subjects were included in the effectiveness evaluation analysis, excluding 207 subjects missing the final evaluation or unable to be evaluated or missing the effectiveness evaluation item (Pain VAS) (Fig. 1). All 14,543 subjects in the safety evaluation group were administered one tablet (200 mg) of aceclofenac CR once a day during the study period. The mean duration of administration was 42.61 ± 91.90 days (median: 22.00, range: 1.00, 1103.00).

### Basic characteristics of the subjects

66.46% (9666/14,543) subjects were female, and the mean age was 58.78 ± 14.63 years. Depending on the classification of medical treatment, 93.35% (13,569/14,543 subjects) received outpatient treatment. The mean duration of the disease was 1.91 ± 3.28 years, and the purposes of administration of aceclofenac CR were estimated in the order of ‘osteoarthritis’ 38.44% (5590/14,543 subjects), ‘lumbago’ 30.74% (4471/14,543 subjects), and ‘scapulohumeral periarthritis’ 15.26% (2219/14,543 subjects). We found that 23.57% (3428/14,543 subjects) had concurrent diseases, and a total of 5276 diseases were accompanied (Table 1). A 45.26% (6582/14,543 subjects) received concurrent medication, and a total of 13,392 medications were administered. All 14,453 subjects underwent a single dose of aceclofenac CR 200 mg daily during the study period. The mean duration of treatment was 42.60 ± 91.90 days (median: 22.00, range: 1.00, 1103.00).

**Table 1:** Basic characteristics of the study subjects.

Basic characteristics	Number of subjects (percentage)
	(N = 14,543)
	n (%)
**Gender**	
Male	4877 (33.54)
Female	9666 (66.46)
**Age (years)**	
Mean ± SD	58.78 ± 14.63
Median	59.00
Min, max	13.00 (100.00)
Under 19	38 (0.26)
19–30	434 (2.98)
30–39	1090 (7.50)
40–49	2042 (14.04)
50–59	3690 (25.37)
60 and over	7249 (49.85)
**Concurrent disease**	
Hepatic disorder	116 (0.80)
Renal impairment	45 (0.31)
Gastrointestinal disorder	1098 (7.55)
Allergy	79 (0.54)
Other medical histories	2594 (17.84)
**Weight (kg)**	
Mean ± SD	61.86 ± 10.13
Median	61.00
Min, max	30.00 (110.00)
≥ 61 kg	4904 (51.02)
< 61 kg	4708 (48.98)
**Medical classification**	
Inpatient	453 (3.12)
Outpatient	13,569 (93.35)
Inpatient/outpatient combination	513 (3.53)
**Purpose of aceclofenac CR administration**^**a**^	
Rheumatoid arthritis	864 (5.94)
Pain caused by nonarticular rheumatism	492 (3.38)
Ankylosing spondylitis	426 (2.93)
Osteoarthritis	5590 (38.44)
Scapulohumeral periarthritis	2219 (15.26)
Lumbago	4471 (30.74)
Ischiadynia	1580 (10.86)
**Duration of the disease (years)**	
Mean ± SD	1.91 ± 3.28
Median	0.58
Min, max	0.00 (40.42)

*SD* standard deviation, *CR* controlled release.

^a^Multiple counting.

### Safety of aceclofenac CR

Among the 14,543 patients, 143 AEs were reported in 125 subjects [0.86%, 95% C.I. (0.72, 1.02)], and of these, 121 adverse drug reactions were reported in 107 subjects [0.74%, 95% C.I. (0.60, 0.89)]. There were no serious AEs during this study period. Of the 143 cases, unexpected AEs, such as 'bronchopneumonia' (0.01%), and unexpected adverse drug reactions, were not observed (Table 2). The most common reported AEs were gastrointestinal disorders, such as heartburn and gastrointestinal disorders (66/14,543 subjects, 73 cases). Among them, subjects of 0.37% (54/14,543, 59 cases) were recorded as adverse drug reactions (ADRs) (Supplemental Table 1). As a result of the multivariate factor analysis on these factors, female [OR: 0.624, 95% C.I. (0.412, 0.945)], inpatient treatment [outpatient *vs*. inpatient OR: 0.330, 95% C.I. (0.183, 0.596), inpatient/outpatient combination *vs*. inpatient OR: 0.138, 95% C.I. (0.031, 0.617)], accompanying concurrent diseases [OR: 2.798, 95% C.I. (1.942, 4.030)], and receiving concomitant drugs [OR: 2.457, 95% C.I. (1.642, 3.678)] were found to have a relatively high incidence of AEs (*P* = 0.0259, *P* = 0.0004, *P* < 0.0001 and *P* < 0.0001) (Table 3).

**Table 2:** The occurrence status of adverse events and adverse drug reactions of aceclofenac CR by System Organ Class.

System Organ Class/preferred term^a^	Adverse event			Adverse drug reaction^b^		
	Incidence rate		Number of occurrences	Incidence rate		Number of occurrences
	n	(%)	Cases	n	(%)	Cases
Total adverse event/adverse drug reaction	125	(0.86)	143	107	(0.74)	121
Serious adverse event/adverse drug reaction	0	(0.00)	0	0	(0.00)	0
**Gastrointestinal system disorders**	66	(0.45)	73	54	(0.37)	59
Heartburn	28	(0.19)	28	24	(0.17)	24
Gastrointestinal disorder	13	(0.09)	13	12	(0.08)	12
Abdominal pain	7	(0.05)	7	6	(0.04)	6
Constipation	6	(0.04)	6	2	(0.01)	2
Dyspepsia	3	(0.02)	3	2	(0.01)	2
Nausea	2	(0.01)	2	2	(0.01)	2
Erosive gastritis	2	(0.01)	2	2	(0.01)	2
Indigestion	2	(0.01)	2	2	(0.01)	2
Gastritis	2	(0.01)	2	2	(0.01)	2
Gastrointestinal distress	2	(0.01)	2	2	(0.01)	2
Stomatitis	1	(0.01)	1	1	(0.01)	1
Abdominal discomfort	1	(0.01)	1	1	(0.01)	1
Diarrhea	1	(0.01)	1	1	(0.01)	1
Reflux esophagitis	1	(0.01)	1	0	(0.00)	0
Gastroesophageal reflux	1	(0.01)	1	0	(0.00)	0
Dry mouth	1	(0.01)	1	0	(0.00)	0
**Urinary system disorders**	20	(0.14)	20	20	(0.14)	20
Face edema	20	(0.14)	20	20	(0.14)	20
**Body as a whole-general disorders**	20	(0.14)	20	20	(0.14)	20
Edema	9	(0.06)	9	9	(0.06)	9
Generalized edema	5	(0.03)	5	5	(0.03)	5
Edema legs	2	(0.01)	2	2	(0.01)	2
Asthenia	2	(0.01)	2	2	(0.01)	2
Hot flushes	1	(0.01)	1	1	(0.01)	1
Fever	1	(0.01)	1	1	(0.01)	1
**Central and peripheral nervous system** disorders	9	(0.06)	10	7	(0.05)	8
Dizziness	4	(0.03)	5	3	(0.02)	4
Headache	3	(0.02)	3	2	(0.01)	2
Burning sensation	1	(0.01)	1	1	(0.01)	1
Vertigo	1	(0.01)	1	1	(0.01)	1
**Skin and appendages disorders**	7	(0.05)	8	5	(0.03)	6
Pruritus	2	(0.01)	2	2	(0.01)	2
Rash	2	(0.01)	2	2	(0.01)	2
Aggravated pruritus	1	(0.01)	1	0	(0.00)	0
Urticaria	1	(0.01)	1	0	(0.00)	0
Allergic dermatitis	1	(0.01)	1	1	(0.01)	1
Skin eruption	1	(0.01)	1	1	(0.01)	1
**Respiratory system disorders**	4	(0.03)	4	3	(0.02)	3
Respiratory failure	2	(0.01)	2	2	(0.01)	2
Bronchopneumonia	1	(0.01)	1^c^	0	(0.00)	0
Breath shortness	1	(0.01)	1	1	(0.01)	1
**Psychiatric disorders**	3	(0.02)	3	1	(0.01)	1
Sleep disorder	1	(0.01)	1	0	(0.00)	0
Depression	1	(0.01)	1	0	(0.00)	0
Somnolence	1	(0.01)	1	1	(0.01)	1
**Liver and biliary system disorders**	1	(0.01)	1	1	(0.01)	1
Liver function tests abnormality	1	(0.01)	1	1	(0.01)	1
**Musculoskeletal system disorders**	1	(0.01)	1	1	(0.01)	1
Non-inflammatory joint swelling	1	(0.01)	1	1	(0.01)	1
**Metabolic and nutritional disorders**	1	(0.01)	1	1	(0.01)	1
Weight increase	1	(0.01)	1	1	(0.01)	1
**Vision disorders**	1	(0.01)	1	1	(0.01)	1
Diplopia	1	(0.01)	1	1	(0.01)	1
**Cardiovascular disorders**	1	(0.01)	1	0	(0.00)	0
High blood pressure	1	(0.01)	1	0	(0.00)	0

^a^Some patients had more than one adverse event.

^b^The causal relationship with the use of aceclofenac CR is certain, probable, possible, conditional/unclassified, or unassessable.

^c^Unexpected adverse event.

**Table 3:** Factors affecting the development of adverse event occurrence (multivariate factor analysis).

Factor	Estimate	Standard error	Odds Ratio		*P*-value
			Estimate	95% CI	
Gender (1 = male, 0 = female)	− 0.471	0.211	0.624	[0.412,0.945]	0.0259
**Medical classification**					0.0004
(1 = outpatient, 0 = inpatient)	− 1.108	0.301	0.330	[0.183,0.596]	
(1 = inpatient/outpatient combination, 0 = inpatient)	− 1.980	0.764	0.138	[0.031,0.617]	
Concurrent disease (1 = yes, 0 = no)	1.029	0.186	2.798	[1.942,4.030]	< 0.0001
Concomitant drug (1 = yes, 0 = no)	0.899	0.206	2.457	[1.642,3.678]	< 0.0001

95% CI = 95% confidence interval.

Multiple logistic regression analysis.

### Effectiveness of aceclofenac CR

The effectiveness analysis was performed on 14,336 patients out of the 14,543 safety evaluation subjects, excluding 32 subjects missing evaluation, 128 subjects unable to be evaluated, and 47 subjects missing VAS measurement. The effectiveness evaluation was performed in 4 scales (recovered, improved, unchanged, and worsened) at the last visit, carefully considering changes in the 10 cm-VAS, subjective and objective symptoms. Signs at 4 weeks after the administration of aceclofenac CR tablet compared to pre-administration, ‘recovered’ and ‘improved’, were defined as ‘effective’, and ‘unchanged’ and ‘worsened’ were defined as ‘ineffective’. Four weeks after the administration of aceclofenac CR, the VAS pain decreased significantly by 2.95 ± 1.83 cm (median: − 3.00, range: − 9.00, 5.00) compared to pre-administration (*P* < 0.0001) (Table 4). The clinical efficacy evaluation showed that ‘recovered’ and ‘improved’ were 12.49% (1790/14,336 subjects) and 79.14% (11,346/14,336 subjects), respectively, with effective rate of 91.63% (13,136/14,336 subjects).

**Table 4:** Effectiveness of aceclofenac CR (N = 14,336).

Pain intensity (10 cm-VAS)	Mean ± SD	Median	*P*-value
Before administration	6.13 ± 1.59	6.00	
4 weeks after administration	3.18 ± 1.47	3.00	
Change^a^	− 2.95 ± 1.83	− 3.00	< 0.0001^b^

Clinical effectiveness^c^	n	(%)	
**Effective**	13,136	(91.63)	
Recovered	1790	(12.49)	
Improved	11,346	(79.14)	
**Ineffective**	1200	(8.37)	
Unchanged	1092	(7.62)	
Worsened	108	(0.75)	

*CR* controlled release, *VAS* visual analogue scale, *SD* standard deviation.

^a^Change = 4 weeks after administration – before administration.

^b^Wilcoxon signed-rank test.

^c^Recovered: All signs and symptoms of pain have disappeared, and no further treatment is deemed necessary, Improved: Signs and symptoms of pain are judged to be significantly improved, Unchanged: No changes in signs and symptoms of pain are observed, and Worsened: Signs and symptoms of pain recurred or deteriorated.

## Discussion

This study was conducted to investigate the safety and effectiveness of patients treated with aceclofenac CR to treat pain and inflammation under the actual condition of administration after marketing this drug. In addition, this study was carried out to identify differences in the incidence and occurrence of adverse cases according to the basic information of each patient.

The incidence of AEs was 0.86%, and there were no significant AEs in this study. The incidence of ADRs was 0.74%, and of these, the most frequent ADRs were gastrointestinal disturbances such as heartburn and gastrointestinal disorders with 0.37%. Although 1 case of ‘bronchopneumonia’ was collected as an unexpected AE, it was not an ADR, and the patient recovered after appropriate treatment. These results could confirm the incidence and severity of AEs related to aceclofenac CR in routine clinical practice settings. In addition, it was shown that its ADRs exceeding previously reported safety information on the existing immediate-release formulation of aceclofenac were not observed^14,28–30^.

In this study, we tried to reaffirm the incidence of ADRs, which were known as general side effects of NSAID but reported less than 1% incidence rate as an analysis result of a conventional immediate-release formulation of aceclofenac in the post-marketing pharmacovigilance study conducted for 1 year in the United Kingdom^14^. As a result of this study for aceclofenac CR, ‘gastrointestinal bleeding’ and ‘abdominal pain’ were not observed similar to those of the previous post-marketing surveillance for an existing aceclofenac immediate release. In the case of ‘hypertension’, 1 case (‘high blood pressure’) occurred in this study. However, it was a temporary case that the causal relationship with aceclofenac CR was evaluated as ‘low probability’, as other medications or potential diseases may reasonably explain it. The incidence of ‘hepatotoxicity’ was observed as ‘liver function tests abnormality’ with one case in this study, which showed a lower incidence rate than those of hepatotoxicity with 0.241% in the previous post-marketing study for conventional aceclofenac formulation. This incidence frequency was also lower than the average incidence rate of 2.5% for hepatotoxicity induced by other NSAIDs such as diclofenac, indomethacin, and naproxen^18,29–32^.

In this study, nephrotoxicity or thromboembolic cardiovascular side effects, which also showed low observations in the same previous surveillance, did not occur for aceclofenac CR^5^. However, regular administration of NSAIDs increases risk of cardiovascular and renal complications^33^. Especially, the AEs of NSAIDs are the consequences of inhibiting prostaglandin synthesis, resulting in acute renal failure. Short-term NSAID administration for up to 6 weeks showed renal reversible AEs^34^ whereas long-term NSAID therapy for 12 months induced reversible or irreversible renal damage as determined by measuring glomerular filtration rate (eGFR)^35^. In addition, previous studies have demonstrated that the risk profiles of AEs are different for each NSAID^36^. Therefore, our study showing no renal AEs might be attributed to the short duration time for 4 weeks and the low renal risk of conventional aceclofenac. In this clinical trial, however, there are no laboratory data about creatinine or eGFR, and further studies are needed.

We evaluated the difference in the incidence of AEs according to the basic information of each patient. The incidence rate of AEs according to gender was relatively high in females, but it was showed a low incidence in both genders with less than 1%. Although the incidence rate of AEs was significantly higher in subjects with concurrent disease than in subjects without concurrent disease, it is also expected to show a relatively similar pattern above when considering each incidence rate. This is because the incidence rate of AEs is expected to be higher in inpatients, owing to the close observation during hospitalization. In inpatient treatment, the underlying diseases were in a relatively worsened state. In addition, the incidence of AEs was significantly increased in subjects receiving concomitant medication. For example, The nephrotoxicity of combinations of NSAIDs with renin–angiotensin signaling inhibitors and/or diuretics induced a high incidence of AEs^37^. Still, it cannot be concluded that the predecessor drug had an AE on the occurrence of AEs. Therefore, careful interpretation is required since there would be many confounding factors and limitations to confirm factors affecting the AEs that occurred in the observational study's nature^38,39^.

The effectiveness of aceclofenac CR was evaluated with careful consideration, including the VAS pain at 4 weeks after using this drug. The effective clinical rate was shown in 91.63% of all the study subjects. These results assured the effectiveness of aceclofenac CR for the treatment of pain and inflammation in routine clinical practice settings. Aceclofenac is an NSAID with a relatively high COX-2 selectivity. COX-2 is a cytokine or a specific signal induced by signal stimuli and increased expression in inflamed tissues. At the same time, COX-1 is primarily expressed in normal tissues and functions to maintain the homeostasis of cells or tissues. Aceclofenac has been reported that the inhibitory effect of COX-1, which causes gastrointestinal toxicity, is relatively low, and the low incidence of adverse cases including gastrointestinal disturbances due to site-specific inflammation^5,6,9,13,22,40,41^.

A sufficiently large sample is needed for an appropriate safety evaluation of AEs that rarely occur in clinical studies to confirm the high probability of occurrence of adverse reactions^42,43^. This study is meaningful because it proved among a large scale of subjects. This study can be provided as a reassessment of the safety in the everyday care environment as an AE not occurring in a controlled trial of a limited number of relatively healthy patients. Furthermore, the results of this study assure the safety and effectiveness of aceclofenac CR as a sustained-release formulation that improves adherence to medication and efficacy by taking advantage of its fast-acting properties due to this drug is a once-daily tablet with 200 mg of aceclofenac.

However, there are some limitations in this study. First, the present study is limited only to outpatients receiving once daily aceclofenac CR during 4 weeks. As the incidence and severity of AEs should be coupled to the duration of intake, further studies for long-term safety and efficacy evaluations are required to clarify these results. Second, the included pain types and clinical syndromes are extremely diverse in this trial. As concomitant medications also biase or influence our findings, the interpretation should be cautious. Therefore, further analysis taking into account various types of syndromes and concomitant medications might be necessary.

## Conclusions

In conclusion, this study could confirm the incidence of adverse events (AEs) and effective clinical rate for treating pain and inflammation associated with aceclofenac CR in the clinical practice environment. Adverse reactions that exceeded the previously reported safety results were not observed. These results of analyzing the prescription status, AE occurrence and pattern, and effective rate are believed to be usefully utilized as references for clinical treatment and the relevant drugs in the future.

## Supplementary Information

Supplementary Table 1.
