# Supplementary material for: Safety and effectiveness of 4-week therapy with aceclofenac controlled release once a day
Source: Sci Rep. 2022 Oct 3;12:16519. doi: 10.1038/s41598-022-20633-6 (PMC9530112; doi:10.1038/s41598-022-20633-6)
Supplement: Supplementary file 1 — Supplementary Table 1. [file 41598_2022_20633_MOESM1_ESM.docx]

Supplementary Table 1. The occurrence status of adverse events depending on the basic information.

| Factor | Classification | Adverse events | | | Total | | *P*-value^a^ |
| --- | --- | --- | --- | --- | --- | --- | --- |
|  |  | n | (%) | 95% CI | n | (%) |  |
| Gender | | | | | | | |
|  | Male | 30 | (0.62) | [0.42,0.88] | 4,877 | (33.54) | 0.0233 |
|  | Female | 95 | (0.98) | [0.80,1.20] | 9,666 | (66.46) |  |
| Age (years) | | | | | | | |
|  | Under 19 | 1 | (2.63) | [0.07,13.81] | 38 | (0.26) | 0.1017 |
|  | 19-30 | 1 | (0.23) | [0.01,1.28] | 434 | (2.98) |  |
|  | 30-39 | 4 | (0.37) | [0.10,0.94] | 1,090 | (7.50) |  |
|  | 40-49 | 23 | (1.13) | [0.72,1.69] | 2,042 | (14.04) |  |
|  | 50-59 | 28 | (0.76) | [0.50,1.09] | 3,690 | (25.37) |  |
|  | 60 and over | 68 | (0.94) | [0.73,1.19] | 7,249 | (49.85) |  |
| Weight | | | | | | | |
|  | ≥61kg | 44 | (0.90) | [0.65,1.20] | 4,904 | (51.02) | 0.6846 |
|  | <61kg | 46 | (0.98) | [0.72,1.30] | 4,708 | (48.98) |  |
| Medical classification | | | | | | | |
|  | Inpatient | 13 | (2.87) | [1.54,4.86] | 453 | (3.12) | 0.0002 |
|  | Outpatient | 110 | (0.81) | [0.67,0.98] | 13,569 | (93.35) |  |
|  | Inpatient/Outpatient combination | 2 | (0.39) | [0.05,1.40] | 513 | (3.53) |  |
| Purpose of aceclofenac CR administration^b^ | | | | | | | |
|  | Rheumatoid arthritis | 15 | (1.74) | [0.97,2.85] | 864 | (5.94) |  |
|  | Pain caused by nonarticular rheumatism | 12 | (2.44) | [1.27,4.22] | 492 | (3.38) |  |
|  | Ankylosing spondylitis | 4 | (0.94) | [0.26,2.39] | 426 | (2.93) |  |
|  | Osteoarthritis | 52 | (0.93) | [0.70,1.22] | 5,590 | (38.44) |  |
|  | Periarthritis of scapulohumerus | 20 | (0.90) | [0.55,1.39] | 2,219 | (15.26) |  |
|  | Lumbago | 43 | (0.96) | [0.70,1.29] | 4,471 | (30.74) |  |
|  | Ischiadynia, | 14 | (0.89) | [0.49,1.48] | 1,580 | (10.86) |  |
| Gastrointestinal disorder | | | | | | | |
|  | Yes | 27 | (2.46) | [1.63,3.56] | 1,098 | (7.55) | <0.0001 |
|  | No | 98 | (0.73) | [0.59,0.89] | 13,445 | (92.45) |  |
| Concurrent disease | | | | | | | |
|  | Yes | 66 | (1.93) | [1.49,2.44] | 3,428 | (23.57) | <0.0001 |
|  | No | 59 | (0.53) | [0.40,0.68] | 11,115 | (76.43) |  |
| Concomitant drug | | | | | | | |
|  | Yes | 90 | (1.37) | [1.10,1.68] | 6,582 | (45.26) | <0.0001 |
|  | No | 35 | (0.44) | [0.31,0.61] | 7,961 | (54.74) |  |

95% CI=95% confidence interval

^a^ Pearson's chi-square test or Fisher's exact test

^b^ *P*-value was not analyzed due to multiple counting
